# Translation and cross-cultural adaptation of the 2017 Oncology Nurse Navigator Core Competencies in Brazil

**DOI:** 10.31744/einstein_journal/2025AO1348

**Published:** 2025-07-11

**Authors:** Mariana Lucas da Rocha Cunha, Alexandre de Souza Barros, Eduarda Ribeiro dos Santos

**Affiliations:** 1 Faculdade Israelita de Ciências da Saúde Albert Einstein Hospital Israelita Albert Einstein São Paulo SP Brazil Faculdade Israelita de Ciências da Saúde Albert Einstein, Hospital Israelita Albert Einstein, São Paulo, SP, Brazil.; 2 Faculdade Israelita de Ciências da Saúde Albert Einstein Hospital Israelita Albert Einstein São Paulo SP Brazil Academic, Faculdade Israelita de Ciências da Saúde Albert Einstein, Hospital Israelita Albert Einstein, São Paulo, SP, Brazil.

**Keywords:** Patient navigation, Nurses, Neoplasms, Professional competence, Disease management

## Abstract

**Objective:**

This study aimed to translate and cross-culturally adapt the Oncology Nurse Navigator Core Competencies 2017 instrument into Brazilian Portuguese.

**Methods:**

This study used a methodological cross-cultural adaptation approach. After authorization from the authors of the original version of the Oncology Nurse Navigator Core Competencies 2017 comprising 52 competencies, the instrument was translated following the recommended guidelines. A panel of five oncology specialists evaluated and consolidated the translation. To analyze the adequacy of the translation, 59 nurse judges—all oncology nurse navigators—were recruited using the snowball technique. Data was collected between December 2020 and August 2022.

**Results:**

The first and second rounds of assessing the suitability of the translated competencies involved 24 and 17 judges, respectively. Their average age was 37.5 years and the majority (87.5%) were women. After incorporating the requested adjustments based on feedback from the second round, the questions exhibited satisfactory proportions of agreement and content validity ratios.

**Conclusion:**

The 52 competencies in 5 categories were adapted and adequate according to the proportions of agreement and content validity ratios, which assumed values compatible with those suggested for adequacy. We therefore conclude that these competencies enable a better understanding of the role and performance of oncology nurse navigators in the Brazilian context.

## In Brief

Cunha et al. translated and adapted the Oncology Nurse Navigator Core Competencies 2017 into Brazilian Portuguese through a validated cross-cultural process. Following an expert review and two evaluation rounds with oncology nurses, all its 52 competencies were deemed suitable, enhancing the understanding of the role of oncology nurse navigators in Brazil.

## Highlights

■ The 52 Oncology Nurse Navigator Core Competencies, across five categories, were cross-culturally adapted.

■ The competencies outline oncology nurse navigator’s role in Brazil.

## Graphical abstract

**Figure:**
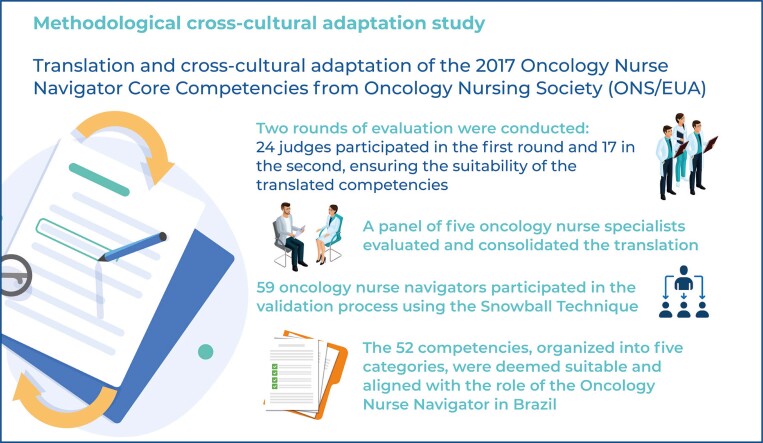

## INTRODUCTION

The role of oncology nurse navigators (ONNs) initially reflected the aim of being a professional who possesses knowledge, skills, and clinical experience and is responsible for providing focused care to patients and their families, considering emotional, physical, and social aspects. Nowadays, patient navigators aim to guide people to overcome socioeconomic, financial, cultural, psychological, and bureaucratic barriers that can hinder access to health services and systems.^1^

This model of care was proposed by American doctor Harold Freeman in 1990 at the Harlem Hospital Center in New York City, in partnership with the American Cancer Society. It aimed to not only expedite confirmation of the diagnosis of people with or suspected of having cancer but also guarantee continuity of treatment from start to finish. In the idealization of the navigator concept, other professionals such as social workers, as well as the lay public, could essay this role. However, owing to their training, nurses can effectively navigate patients through the health system, accumulating the necessary clinical oncology knowledge.^1,2^

In 2012, the Oncology Nursing Society (ONS) conducted the ONN Role Delineation Study, with the aim of examining the activities and knowledge required for the role, avoiding overlaps between navigators and clinicians.^3^

After a thorough review of the literature, the ONS defined an ONN as: “A nurse with specific clinical expertise in the field of oncology, whose role is to provide individualized care to patients, family and caregivers to help overcome barriers in health care systems, based on the nursing process. Oncology Nursing Society educate and provide resources that facilitate information-based decision-making and timely access to quality health care and psychological care throughout all stages of the cancer disease process.”^3^

In 2013, the ONS outlined the Oncology Nurse Navigator Core Competencies, reviewing and updating their scope in 2017^4,5^ to include a new category with 12 competencies for specialist nurses, thereby establishing a list of 52 essential competencies that ONNs are expected to possess.^5-7^

Patient navigation is perceived by many as a key component of treatment and support for cancer patients of any population,^8^ despite most studies focusing on those with limited financial, social, and health resources.^9,10^

Studies have revealed that navigation can help reduce the time between diagnosis and the first consultation and/or treatment, promote resource optimization, and ensure rapid referral to mitigate barriers.^11,12^ The literature also contends that navigation can increase patient satisfaction, improve the experience of care, and significantly increase the quality of life assessment scores of patients treated by ONNs.^11-13^ A breast cancer center, located in southern Brazil pioneered the implementation of the first nurse navigation program in 2016, subsequently publishing results that corroborate the positive findings on the role of ONNs.^14^

That same year, the National Supplementary Health Agency (ANSS) launched—through Oncorede—a program called The Reorganization of the Cancer Care Network in Supplementary Health. The document proposed recommendations to implement a navigation program, the basic premise of which was that the system needed to be organized before it could be navigated.^15^

A 2017 study defined the basic duties of the navigator. In this program, the aforementioned principles were consolidated, expanding the involvement of patients and families in achieving goals; considering social, cultural, and cognitive conditions in obtaining resources; and optimizing results through education and collaborating with the multidisciplinary team.^16^

Despite extensive efforts to define the core competencies of ONNs, gaps remain in the understanding of their role and responsibilities,^17^ especially in countries like Brazil, where their work is relatively new and not well established, either in practice or in specific regulations.^18^ Research on navigation in the Brazilian context remains scarce.^16^

Clearly defined ONN competencies can help establish their roles and responsibilities, ensure the proper selection of professionals, evaluate their performance and development, identify their strengths, develop orientation programs to nurture skills, and assess the functioning of the oncology navigation program, among other benefits.^19^

The recognition of the importance of ONNs, together with the need to outline their role in the Brazilian context, contemplating the benefits mentioned above, instigated this research, which aims to translate and cross-culturally adapt the 2017 Oncology Nurse Navigator Core Competencies into Brazilian Portuguese.

Recently, the Federal Nursing Council issued Resolution COFEN No. 735, dated January 17, 2024, establishing standards^20^ pertaining to the role of nurse navigators and defining their duties and requirements. In this sense, the proposal to transculturally adapt the competencies to be developed by ONNs, considering not only the specificities of the Brazilian health system but also the realities of clinical practice and care management can promote progress in the scope of action and validation of this professional role in Brazil.

## OBJECTIVE

This study aimed to translate and cross-culturally adapt the Oncology Nurse Navigator Core Competencies 2017 instrument into Brazilian Portuguese.

## METHODS

This methodological cross-cultural adaptation study was conducted between December 2020 and August 2022. The original English document outlining the Oncology Nurse Navigator Core Competencies was revised and updated to include 52 competencies, theoretically divided into 5 categories: coordination of care, communication, education, role of the professional, and ONN-specialist, with 13, 9, 11, 7, and 12 competencies, respectively.^5-7^

Permission for the adaptation was obtained via e-mail from the ONS. The adaptation process was carried out according to the guidelines of the Recommendations for the Cross-Cultural Adaptation of the DASH & QuickDASH Outcome Measures.^21^ It was divided into two phases: translate and cross-culturally.

### Stage 1: Translation

**Figure 1:**
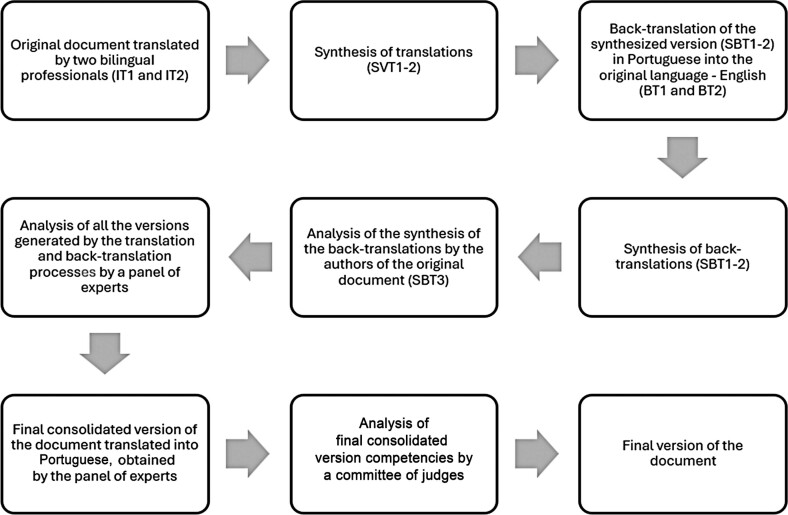
Translation and cross-cultural adaptation process of the 2017 Oncology Nurse Navigator Core Competencies

IT1: Independent translation 1; IT2: Independent translation 2; SVT1-2: Synthesis of translations; BT1: Backtranslation 1; BT2: Backtranslation 2; SBT1: Synthesis of back-translation 1; SBT2: Synthesis of back-translation 2; SBT3: Synthesis of the back-translations by the authors of the original document; SBT1-2: Synthesis of back-translations.

For a better understanding of the translation and adaptation process, the stages have been summarized in a flowchart (Figure 1).

Two bilingual professionals independently carried out two translations (IT1 and IT2). The first translator (T1) is a proofreader and nurse with a bachelor’s degree in International Relations from Boston University. The second translator (T2) has a master’s degree in English and is a tutor in an academic literacy laboratory. After the translations, the main researcher and supervisors analyzed the translated versions, considering linguistic aspects and the meanings given to the terms in Brazilian reality, generating a synthesized version (SVT1-2).

This synthesized version in Portuguese (SVT1-2) was back-translated into the original language—English—by two independent translators who were native English speakers and proficient in Portuguese, generating back-translated versions 1 and 2 (BT1 and BT2). Both these versions were analyzed and synthesized by the researcher and supervisors to identify possible discrepancies, creating a new English version (SBT1-2). This version (SBT1-2) was then sent to the authors of the original document to not only assess the English translation but also identify any conceptual dissonances, finally generating the version called synthesis of back-translations 3 (SBT3).

Following the assessment of the synthesis of back-translations by the authors of the original document, all the versions produced—IT1 and IT2, SVT1-2, VBT1 and VBT2, SBT1-2, and SBT3—were analyzed by a panel of experts to reconcile discrepancies with the original document, resolve doubts, and provide recommendations to obtain a consolidated translation of the list of competencies. This evaluation was conducted in a virtual meeting between the researchers and experts, which involved analyzing all the translations, competency by competency, until the consolidated version—the final translated version (FTV)—was completed.

The panel of experts for this analysis comprised five nurses with clinical experience in cancer care and fluent in the original language of the document (English). Among them, one had a doctorate and two had a master’s degree in nursing, one had a master’s degree in oncology, and one was an oncology specialist. They consented to participating in the research by signing an informed consent form.

### Stage 2: Cross-cultural adaptation

The second phase of the research involved the evaluation of the final consolidated version (FCV) by a group of judges.

### Study population

This study consisted of oncology nurses, referred to as judges. To select the sample, we used the snowball technique and key informants, known as “seeds,” to locate individuals with the profile required for the research, from their own personal network. Jasper’s criteria were also considered^22^ such as knowledge and skills, practical experience, and recognition by others. The judges had to (1) be in the nursing profession, working in main hospital units providing specialized high-complexity care; (2) have at least one year of experience in the field of oncology; (3) have worked as an ONN; and (4) not have stepped away from the profession for more than one year.

### Data collection

The data for the second phase of the survey were collected between June and August 2022, using individual forms designed by the authors and sent electronically to the judges. The instrument consisted of two parts, the first containing data on judges’ characteristics and the second containing the translated ONN core competencies.

The document was submitted for evaluation by the judges, who indicated whether they agreed with the competencies or suggested changes. The competencies were rated on a Likert-type scale with the following options: “strongly agree,” “agree,” “disagree,” or “strongly disagree.” When judges selected “disagree” or “strongly disagree,” an automatic option appeared to select “keep with adjustments,” followed by a field to suggest adjustments or t the option to exclude the competency. Participants in the second phase received an online free and informed consent form and, after agreeing to participate, were automatically directed to the response form.

### Data analysis

For the answers obtained in the second phase by the judges in relation to the suitability of the competencies, the proportions of agreement (agree + strongly agree) were calculated, and competencies with a proportion of agreement of less than 80% were selected for adjustment. Once the selected competencies were adjusted, they were sent back to the judges for assessment regarding their suitability. In addition to the proportions of agreement, the content validity ratios for each competency were calculated, and the obtained values were compared with the minimum values recommended by Ayre et al,^23^ which vary according to the total number of judges, to draw conclusion about their adequacy.

Agreement between the judges’ responses was assessed using Gwet’s AC1 agreement coefficient, with their respective 95% confidence intervals (95%CI). The coefficients were considered satisfactory if greater than 0.6 and excellent if greater than 0.8, according to Altman.^24^ The minimum number of judges previously defined for the analysis was nine, as recommended in the literature.^25^ Analyses were conducted using the R, irrCAC and SPSS packages, and data was collected using the REDCap platform.

This study complied with the rules of Resolution 466 of December 12, 2012, of the National Health Council. Data collection occurred after approval by the Research Ethics Committee of *Hospital Israelita Albert Einstein* (CAAE: 35508920.8.0000.0071; # 4.222.112).

## RESULTS

For the assessment of competencies in the second phase of the research, 59 nurse judges were invited, 24 of whom consented to participate in the study and completed the demographic and competency assessment questionnaire. Their average age was 37.5 years, they were predominantly women (87.5%), served primarily as cancer patient navigators (90%), were oncology specialists (90%) and had between one and three years’ experience in the area (58.3%). Although most nurses were from the state of São Paulo (58.3%), some represented the Midwest, Northeast, and Southern regions, totaling nine states.

The data description from the first evaluation by the judges revealed that 44 competencies had a proportion of agreement equal to or greater than 80%. Subsequently, feedback regarding the results obtained was sent to the 24 judges and eight questions were reworded for re-evaluation. The competencies indicated for reformulation in Round 1 are categorized in table 1.

**Table 1:** Competencies identified in the first round of assessment

Category	Competency	Number of answers in agreement/Number of judges	Proportion of agreement (%)
1: Coordination of care	Competency 4: uses assessment tools and methods	19/24	79.2
	Competency 9: makes referrals for molecular and/or genetic testing and counseling	17/23	73.9
	Competency 13: applies knowledge of health insurance company, health insurance and Unified Health System (SUS) processes	17/24	70.8
3: Education	Competency 9: raises awareness of/guidance on clinical research	14/24	58.3
	Competency 11: ensures understanding of molecular and genomic tests	17/24	70.8
5: Oncology nurse navigator–specialist	Competency 5: extends or develops new processes to evaluate indicators and presents them to the service’s leadership	19/24	79.2
	Competency 11: develops strategies necessary to comply with guidelines and recommendations from societies	18/24	75.0
	Competency 12: contributes to the sustainability, improvement, and/or development of the nurse navigator program	15/24	62.5

Regarding the changes made to the coordination of care, in competency 4, nurses were found to be unfamiliar with the specific patient assessment tools adopted in the United States, such as the distress thermometer. In this sense, the adaptation aimed to maintain tools commonly used in Brazil. Competency 9, which deals with molecular and genomic tests and their implications, was adjusted by opting to adapt the role to the facilitation and appropriate referral of the patient. Regarding competency 13, the judges determined that it would not be possible to validate health systems other than those in force in Brazil. The changes were aimed at adapting, for example, to the Brazilian Unified Health System (SUS - *Sistema Único de Saúde*) and the Brazilian supplementary health chain.

The education category underwent adjustments in two competencies, 9 and 11. The former was related to promoting and raising awareness among patients, their families, and caregivers about clinical research because, as per the judges’ understanding, this is not done in Brazil. It was agreed that ONNs would be responsible for promoting or facilitating their access to research. The latter related to the role of ONNs in providing information on molecular and genomic tests and their implications. Changes were needed to ensure that they acquired proper understanding of the subject and did not provide results directly to patients and their families.

With regard to the competencies assigned to ONN specialists, the collection and proposal of indicators (5 and 11) needed to be changed to cancer committees and the leadership of oncology services, in addition to the emphasis on following guidelines and recommendations from Brazilian oncology and/or hematology societies. Competency 12, which deals with the administration, sustainability, improvement, and/or development of navigation programs, as well as requests for funding and philanthropy, was amended by replacing the term “institutional support foundation” with institution.

In this second evaluation, 17 of the 24 initial judges participated. After a new evaluation by the judges, the calculations of the proportion of agreement for the eight altered competencies revealed a value higher than the minimum suggested for 17 judges, which was 76.5^23^(Table 2). Thus, no further changes were necessary. The content validity ratio for the 52 competencies were calculated and all were found to be adequate according to the values observed, which were higher than the cut-off points suggested for each number of judges (Supplementar Material - *Versão em Português*). In addition, the assessment of agreement between the responses of the different judges using Gwet’s AC1 coefficient demonstrated excellent agreement (coefficients greater than 0.80) for all the competencies, with the coefficient for coordination of care being 0.86 (95%CI= 0.78 to 0.93); for communication, 0.99 (95%CI= 0.97 to 1.00); for education, 0.97 (95%CI= 0.92 to 1.00); for role of the professional, 0.90 (95%CI= 0.79 to 1.00); for ONN-specialist, 0.83 (95%CI= 0.77 to 0.88); and, for the grouped competencies, 0.91 (95%CI= 0.88 to 0.94).

**Table 2:** Judges’ evaluation of the adequacy of the competencies after the second round

	Competency	Number of answers in agreement/Number of judges	Proportion of agreement (%)
1: Coordination of Care	Competency 4 ^#^	16/17	94.1
	Competency 9 ^#^	17/17	100.0
	Competency 13 ^#^	16/17	94.1
3: Education	Competency 9 ^#^	15/17	88.2
	Competency 11 ^#^	17/17	100.0
5: Oncology Nurse Navigator–Specialist	Competency 5 ^#^	16/17	94.1
	Competency 11 ^#^	16/17	94.1
	Competency 12 ^#^	15/17	88.2

# Question obtained after changes and evaluation by the judges in round 2.

After the adaptation process, a list of 52 competencies from the 2017 Oncology Nurse Navigator Core Competencies was obtained, translated, and adapted into Brazilian Portuguese (Table 1S, Supplementary Material).

**Table 1S:** Results of the proportions of agreement and content validity ratios obtained for the 52 competencies translated and adapted into Brazilian Portuguese

Competency	Number of answers in agreement/Number of judges	Proportion of agreement	Content validity ratio
1: Coordination of Care			
Question 1	21/24	87.5	0.75
Question 2	23/24	95.8	0.92
Question 3	20/23	87.0	0.74
Question 4 ^#^	16/17	94.1	0.88
Question 5	24/24	100.0	1.00
Question 6	24/24	100.0	1.00
Question 7	22/24	91.7	0.83
Question 8	21/24	87.5	0.75
Question 9 ^#^	17/17	100.0	1.00
Question 10	21/24	87.5	0.75
Question 11	22/24	91.7	0.83
Question 12	23/24	95.8	0.92
Question 13 ^#^	16/17	94.1	0.88
2: Communication			
Question 1	24/24	100.0	1.00
Question 2	24/24	100.0	1.00
Question 3	24/24	100.0	1.00
Question 4	24/24	100.0	1.00
Question 5	23/24	95.8	0.92
Question 6	24/24	100.0	1.00
Question 7	24/24	100.0	1.00
Question 8	24/24	100.0	1.00
Question 9	24/24	100.0	1.00
3: Education			
Question 1	24/24	100.0	1.00
Question 2	24/24	100.0	1.00
Question 3	24/24	100.0	1.00
Question 4	24/24	100.0	1.00
Question 5	23/24	95.8	0.92
Question 6	24/24	100.0	1.00
Question 7	24/24	100.0	1.00
Question 8	24/24	100.0	1.00
Question 9 ^#^	15/17	88.2	0.76
Question 10	24/24	100.0	1.00
Question 11 ^#^	17/17	100.0	1.00
4: The role of the professional			
Question 1	24/24	100.0	1.00
Question 2	23/23	100.0	1.00
Question 3	23/23	100.0	1.00
Question 4	22/24	91.7	0.83
Question 5	21/24	87.5	0.75
Question 6	22/24	91.7	0.83
Question 7	21/22	95.5	0.91
5: Oncology nurse navigator - specialist			
Question 1	21/24	87.5	0.75
Question 2	22/24	91.7	0.83
Question 3	23/24	95.8	0.92
Question 4	23/24	95.8	0.92
Question 5 ^#^	16/17	94.1	0.88
Question 6	20/24	83.3	0.67
Question 7	22/24	91.7	0.83
Question 8	23/24	95.8	0.92
Question 9	23/24	95.8	0.92
Question 10	22/24	91.7	0.83
Question 11 ^#^	16/17	94.1	0.88
Question 12 ^#^	15/17	88.2	0.76

# Competency obtained after changes and second evaluation by the judges.

Minimum proportion of agreement according to the number of judges: 76.5 with 17 judges, 72.7 with 22, 69.6 with 23, and 70.8 with 24; Minimum content validity ratio according to the number of judges: 0.529 with 17 judges, 0.455 with 22, 0.391 with 23, and 0.417 with 24.

## DISCUSSION

The document containing the 52 core competencies for ONNs, divided into five categories, was cross-culturally adapted after two rounds of evaluation by the judges. After incorporating the requested adjustments based on feedback from the second round, the proportions of agreement and content validity ratios showed adequate values, as did the coefficients of agreement between the judges in all categories.

The eight competencies that required adjustments or reformulations, in contrast to what is done in the American context and defined by the ONS, revealed discrepancies related to nurse autonomy, the health system organization, and reference bodies for cancer patient care in Brazil. In the process of cross-cultural adaptation, some limitations and the need to alter instruments or documents are inevitable, since different languages, realities, and cultures are compared in each country.^26^

Some of the adjustments are of simple relevance, such as knowledge of a particular evaluation tool or the role of ONNs in addressing quality indicators. The research was conducted with judges from private and public health institutions, which could potentially interfere with the resources available for ONNs’ work, depending on the service and the location at which the program is implemented. The literature emphasizes that the navigation program should consider care and management practices in accordance with the health service for which it was designed so as to meet the needs of the patients it assists.^16^ Furthermore, in this concept, human, physical, and financial resources play equally important roles.

The role and autonomy of nurses differ between countries for various reasons, from historical to financial or social issues. Nurses in more developed countries improve their skills and perform specialized procedures, taking on some of the medical duties,^27^ thereby justifying the need to adapt competency 9 of category 1 (coordination of care), for example. In this sense, cross-cultural adaptation helps to adapt the specific competency to the Brazilian scenario.

Providing information about molecular and genomic tests and their implications were also competencies that needed to be adapted owing to the limited autonomy of Brazilian nurses in this area (category 1/competency 9 and category 3/competency 11). However, ONNs must ensure that both family members and patients understand the results of these tests when they are requested.^28^

It is noteworthy that the role of nurses in genetics and genomics, as a nursing specialty, was published by the Federal Nursing Council in Resolution No. 577/2018.^29^ As a relatively recent decision, the responsibilities and independence of nurses in this area are expected to mature with the time needed for recognition by their professional colleagues, the interdisciplinary team, and the community.

The American health system—as well as specialized bodies, foundations, and societies—differs from the Brazilian one, necessitating the adaptation of competencies 13 (category 1), 11, and 12 (category 5). The change did not alter the rationale of the nurses’ role itself but was based on the inclusion of the different models and bodies that focus on restoring, promoting, and protecting health in Brazil, including the SUS and complementary health care.

The study discovered a need to change three of the ONN-specialist competencies (category 5). Regarding the role of evaluating and controlling the program’s quality indicators (competency 5), despite a simple adjustment, some nurse navigators did not understand this requirement as part of their role. International research has revealed positive results from the implementation of ONN programs in terms of management and continuity of care; acceleration of the patient’s journey through the health system; improved communication, satisfaction, and health education; and reduction of cost,^11-13^ especially in areas where economic resources are scarce.^9,11^ In this sense, monitoring these interventions and proposing improvements must be based on the involvement of the nurse navigator throughout the process of diagnosis, care, and patient education.

The model also requires further study from the perspective of clinical evidence on the role of nurses,^27,30^ which can be promoted through better defined competencies regarding the scope of their work. In Brazil, after a little more than five years of implementation of the first navigation programs in public and private systems, many services still require more in-depth structuring and organization, the prioritization of training and—above all—a better definition of the role of the nurse navigator. Given this panorama, the transcultural adaptation of the ONN core competencies to Brazilian reality is essential. The literature reveals that the lack of clarification of this role leads institutions to promote circumstantial adaptations rather than the professionalization of the navigator’s duties.^28,31^

In September 2022, Law 14.450 was passed, creating the National Patient Navigation Program for People with Malignant Breast Neoplasms.^32^ Its main objectives were rapid diagnosis and timely treatment. The need for the cross-cultural adaptation of the ONN competencies was urgent, since the consolidation of this role has been discussed, studied, and implemented in cancer services in Brazil. It is also justified by the need for standardization and qualification, established by the resolution recently defined by COFEN, aimed at ensuring that nurse navigators are equipped with the necessary training and skills to provide comprehensive and efficient care so as to improve patients’ experience of the health system and contribute to more positive clinical outcomes.^20^

The number of nurses working as navigators in the country is still limited, and more than 50% of ONNs nominated for this research chose not to participate in this study. Moreover, approximately one-third of the participants in the first round did not continue with the study, which can be considered a limitation of this research.

## CONCLUSION

The Nurse Navigator Core Competencies were adapted to Brazilian Portuguese, given that, after two rounds of evaluation by the judges, all of them obtained satisfactory proportions of agreement and content validity ratios.

The study demonstrates support for the use of the competencies, enabling a better understanding of the role and performance of Oncology Nursing Society in the Brazilian context. It not only outlines the practical responsibilities of nurse navigators based on essential competencies for this role in the care, educational, and management spheres within the Brazilian context but also contributes to the professionalization of this field of work.

## SUPPLEMENTARY MATERIAL

Translation and cross-cultural adaptation of the 2017 Oncology Nurse Navigator Core Competencies in Brazil
Mariana Lucas da Rocha Cunha, Alexandre de Souza Barros, Eduarda Ribeiro dos Santos
**DOI: 10.31744/einstein_journal/2025AO1348**
**Versão em Português das Competências Essenciais do Enfermeiro Navegador de Oncologia**
**Declaração de Competências: Declaração Introdutória**
O enfermeiro navegador em oncologia (ENO) demonstra pensamento crítico e uso dos processos de enfermagem para avaliar e atender a todas as necessidades dos pacientes e familiares/cuidadores por meio da coordenação da assistência continuada ao câncer. O ENO atua entre os domínios paciente, unidade familiar e sistema de saúde para melhorar a saúde, tratamento e resultados de fim de vida. Tais atividades são conduzidas por meio de práticas competentes nas seguintes áreas funcionais:
**Competência Categoria 1: Coordenação do Cuidado**
O ENO facilita a prestação adequada e eficiente de serviços de saúde tanto dentro dos sistemas de atendimento à saúde como dentre eles atuando como a principal pessoa de contato que, baseado no atendimento centrado no paciente, é responsável pela promoção dos melhores desfechos de saúde. O ENO:
**Table:** 

ENO iniciante
1. Avalia as necessidades do paciente no encontro inicial e em consultas periódicas durante a navegação; aborda demandas não atendidas de forma apropriada por meio de encaminhamento e serviços de suporte, tais como cuidados paliativos, necessidades nutricionais, atendimento médico, serviço social, reabilitação, suporte jurídico e financeiro.
2. Identifica as dificuldades possíveis ou concretas ao acesso aos cuidados de saúde (Ex.: transporte, cuidados oferecidos à população infantil e geriátrica, habitação, idioma, cultura, grau de alfabetização, desigualdade de papéis, aspectos psicossociais e financeiros, situação laboral e seguro saúde), além de facilitar os encaminhamentos necessários para a superação dessas dificuldades.
3. Desenvolve conhecimento em relação aos recursos disponíveis localmente, na comunidade, recursos nacionais e qualidade dos serviços oferecidos, além de estabelecer um relacionamento com os provedores de tais serviços.
4. Desenvolve ou usa ferramentas e métodos de avaliação diagnóstica adequados, tais como escala de dor, escala de fadiga, condições de saúde, entrevistas motivacionais e aspectos financeiros, entre outros, para promover um plano de cuidados consistente e holístico.
5. Facilita de forma eficaz os agendamentos de consultas, exames diagnósticos e procedimentos para definir o plano de cuidado com o intuito de promover a assistência continuada.
6. Participa da coordenação do plano de cuidados com a equipe multidisciplinar, promovendo no momento oportuno as recomendações de tratamento e cuidados de suporte (Ex.: conferências oncológicas e tumor boards).
7. Facilita a assistência individualizada dentro do contexto do estado funcional, considera questões culturais, de alfabetização em saúde, psicossociais, reprodutivas/fertilidade, necessidades espirituais tanto dos pacientes quanto dos familiares e cuidadores.
8. Aplica conhecimento das diretrizes clínicas (Ex.: Rede de câncer nacional, Comitê Conjunto Americano de Câncer) e recursos especiais (Ex.: recursos ONS para inclusão de evidências práticas) ao longo do câncer contínuo.
9. Quando identificados candidatos para teste molecular e/ou testagem e aconselhamento genético, o ENO facilita e/ou orienta encaminhamentos apropriados.
10. Auxilia na transição adequada dos pacientes de tratamento ativo para programa de sobreviventes e gerência de câncer em estágio crônico e cuidados de fim de vida.
11. Auxilia os pacientes oncológicos a lidar com questões relacionadas aos objetivos do tratamento, diretivas antecipadas de vontade, cuidados paliativos e preocupações com o final da vida usando uma estrutura ética isenta de julgamentos ou discriminação.
12. Assegura o registro ou documentação das consultas do paciente e dos serviços realizados.
13. Aplica conhecimento sobre processos de operadoras de planos de saúde, seguro saúde privado, do Sistema Único de Saúde (SUS) e seu impacto no estadiamento, encaminhamentos e decisão de cuidado aos pacientes por meio do estabelecimento de encaminhamentos apropriados, como necessário.
**Competência Categoria 2: Comunicação**
O ENO demonstra habilidades de comunicação interpessoal que possibilitam troca de ideias e de informações de forma efetiva com pacientes, familiares e parceiros em todos os níveis, incluindo habilidades de comunicação escrita, oral e escuta. O ENO:
**Table:** 

ENO iniciante
1. Constrói relação terapêutica e de confiança com os pacientes, familiares e cuidadores por meio da comunicação efetiva e habilidades de escuta.
2. Atua como uma ligação entre os pacientes, seus familiares, cuidadores e provedores de serviços de saúde com o objetivo de otimizar os desfechos.
3. Advoga em favor dos pacientes, promovendo o cuidado centrado no paciente, que inclui tomada de decisão compartilhada sobre o tipo de cuidado que o paciente deseja receber com os melhores desfechos.
4. Providencia apoio psicossocial e facilita encaminhamentos apropriados aos pacientes, familiares e cuidadores, especialmente durante os períodos de alto estresse emocional e ansiedade.
5. Empodera os pacientes e suas famílias para que estes consigam advogar em favor próprio e assim possam transmitir as suas necessidades.
6. Adere às regras estabelecidas no que diz respeito à confidencialidade e privacidade das informações sobre o paciente.
7. Promove um ambiente de cuidado centrado no paciente e sua família para a tomada de decisões éticas e advoga para os pacientes com câncer.
8. Assegura a comunicação culturalmente sensível e apropriada ao nível de letramento em saúde.
9. Facilita a comunicação entre membros das equipes multidisciplinares de assistência oncológica para prevenir a assistência fragmentada ou tardia que pode afetar negativamente os resultados dos pacientes.
**Competência Categoria 3: Educação**
No momento oportuno, o ENO transmite as informações educativas necessárias aos pacientes, seus familiares e cuidadores para que estes, ao tomarem suas decisões, tenham o apoio e o conhecimento necessários.
**Table:** 

ENO iniciante
1. Avalia as necessidades educacionais dos pacientes, seus familiares e cuidadores, levando em consideração as barreiras do cuidado (Ex.: grau de alfabetização, idioma, influências culturais e comorbidades).
2. Providencia e reforça atividades educacionais aos pacientes, familiares e cuidadores sobre o diagnóstico, opções de tratamento, manejo de efeitos adversos, cuidados pós-tratamento e de sobrevivência (Ex.: plano de cuidado de sobrevivência, resumo de tratamento).
3. Educa os pacientes, familiares e cuidadores sobre o papel do ENO.
4. Orienta e educa pacientes, familiares e cuidadores em relação ao sistema de assistência à saúde oncológica, papel da equipe multidisciplinar e recursos disponíveis.
5. Promove a tomada de decisão de forma autônoma dos pacientes por meio do fornecimento de educação e suporte personalizados.
6. Educa e reforça o significado de adesão aos pacientes, familiares e cuidadores em relação à agenda de tratamento, protocolos e acompanhamentos.
7. Avalia e promove escolhas de estilo de vida saudável e estratégias de autocuidado por meio da educação e encaminhamentos de serviços adicionais.
8. Oferece orientação antecipada e administra as expectativas para ajudar os pacientes, familiares e seus cuidadores a lidar com o diagnóstico de câncer e seus desfechos possíveis ou esperados.
9. Promove ou facilita o acesso de pacientes, seus familiares e cuidadores a profissionais que possam efetuar a conscientização/orientação sobre pesquisas clínicas.
10. Obtém ou desenvolve materiais educativos relacionados à área da oncologia a serem distribuídos para os pacientes, equipe e membros da comunidade, conforme apropriado.
11. Assegura o entendimento adequado dos pacientes, seus familiares e/ou cuidadores sobre informações recebidas acerca dos testes moleculares e genômicos, assim como as implicações dos resultados desses testes.
**Competência Categoria 4: Papel do Profissional**
O ENO atua para promover e avançar o papel da sua categoria profissional, sendo também responsável por seu crescimento e desenvolvimento pessoal e profissional. Adicionalmente, o ENO facilita a promoção contínua e melhoria da qualidade do programa de navegação das suas organizações para melhor atender às necessidades da sua comunidade.
**Table:** 

ENO iniciante
1. Incentiva a prática baseada em evidências e se compromete a estudar ao longo de toda a vida para, assim, melhorar o cuidado oferecido aos pacientes com o diagnóstico de câncer, seja no passado, presente ou futuro.
2. Demonstra comunicação efetiva com seus pares, membros da equipe de saúde multidisciplinar, organizações comunitárias e recursos.
3. Contribui para o desenvolvimento, implementação e avaliação do programa do ENO e seu papel dentro do sistema de saúde e da comunidade.
4. Participa do rastreamento e monitoramento de métricas e resultados, em colaboração com a administração para documentar e avaliar resultados do programa de navegação e relatar achados à liderança do serviço de oncologia.
5. Colabora com a liderança do serviço de oncologia e a equipe de administração, realiza e avalia os dados a partir das necessidades da comunidade para identificar áreas de melhoraria que afetarão o paciente no processo de navegação e participa da melhoria da qualidade com base na identificação de lacunas dos serviços.
6. Colabora com outros membros da equipe de assistência à saúde, estabelece parcerias com agências e grupos locais que possam auxiliar no cuidado do paciente com câncer, suporte ou necessidades educacionais.
7. Estabelece e mantém limites profissionais com pacientes, cuidadores e equipe de saúde multidisciplinar em colaboração com gerentes, como definido pela descrição do cargo.
**Competência 5: Enfermeiro Navegador em Oncologia Especialista**
O ENO especialista é proficiente e tem amplo conhecimento do seu papel profissional, possui formação, conhecimento e experiência para aplicação do pensamento crítico e de habilidades de tomada de decisão para oferecer cuidados especializados e realizar a melhoria do processo de navegação.
**Table:** 

ENO especialista
1. Contribui para o desenvolvimento da avaliação das necessidades do programa de oncologia para a comunidade e faz sugestões ao comitê de câncer sobre mudanças no programa de navegação relacionadas aos resultados da avaliação da comunidade e ao plano estratégico do programa de oncologia.
2. Auxilia na análise de lacunas, melhoria de qualidade e medidas de melhoria dos processos, analisa dados e faz recomendações para o comitê de câncer para mudanças apropriadas no programa de navegação relacionada aos dados.
3. Desenvolve e promove caminhos para que o ENO recrute mais pacientes oncológicos por meio de colaborações com parceiros internos e externos.
4. Documenta o uso de recursos internos e externos por colaboradores e pacientes, faz recomendações apropriadas e adota melhorias quando necessário.
5. Amplia os atuais ou desenvolve processos novos para avaliar indicadores (Ex.: nível de satisfação do paciente e/ou cuidador em relação ao serviço de navegação), coleta os resultados desta avaliação e os apresenta para liderança do serviço de oncologia.
6. Contribui para crescimento do programa por meio da colaboração com administração do departamento de oncologia para desenvolvimento de estratégia de *marketing* dando suporte ao programa de navegação.
7. Contribui para a base do conhecimento da comunidade de assistência à saúde como forma de apoiar o papel do ENO, realizando atividades como participação em associações profissionais, apresentações, publicações e pesquisa.
8. Dissemina informações sobre o papel do ENO para os membros de outras equipes de saúde por meio de programas educacionais entre pares, mentorias e preceptorias.
9. Colabora com o(s) médico(s) responsável(veis) e equipe de suporte para prevenir internações ou consultas desnecessárias e melhorar a adesão ao tratamento, elaborando e implementando as devidas medidas educativas e de acompanhamento.
10. Orienta, mentora e guia novos ENOs
11. Colabora com a administração do departamento de oncologia e lideranças do serviço de oncologia para desenvolver estratégias necessárias ao cumprimento de guidelines e recomendações da Sociedade Brasileira de Oncologia e/ou Hematologia.
12. Contribui para a sustentabilidade, melhoria e/ou desenvolvimento do programa de navegação, colaborando com a instituição na elaboração de solicitação de apoio financeiro e/ou filantropia.
